# Exploring biodiversity of Uruguayan vascular plants through DNA barcoding

**DOI:** 10.3389/fgene.2024.1435592

**Published:** 2024-08-21

**Authors:** Cecilia Da Silva, Natalia Mannise, Rosina Seguí, Andrés Iriarte, Nadia Bou, J. Mauricio Bonifacino, Ary Mailhos, Lucía Anza, Santiago Chitaro, Florencia Ocampo, Rosario Gándaras, Florencia Arezo, Leandro Capurro, Marcelo Iturburu, Nicolás Nieto, Hernán Juan, Joaquín Garrido, Raúl Platero, Julián Gago, Felipe Lezama, Martín Do Carmo, Mariana Cosse

**Affiliations:** ^1^ Centro Universitario Regional Noreste, Universidad de La República, Tacuarembó, Uruguay; ^2^ Departamento de Biodiversidad y Genética, Instituto de Investigaciones Biológicas Clemente Estable, Montevideo, Uruguay; ^3^ Laboratorio de Biología Computacional, Departamento de Desarrollo Biotecnológico, Instituto de Higiene, Facultad de Medicina, Universidad de La República, Montevideo, Uruguay; ^4^ División Información Ambiental, DINACEA, Ministerio de Ambiente, Montevideo, Uruguay; ^5^ Facultad de Agronomía, Universidad de La República, Montevideo, Uruguay; ^6^ Facultad de Ciencias, Universidad de La República, Montevideo, Uruguay; ^7^ DNA Barcode Training Course and Grasses Barcode Pilot Project, PEDECIBA Universidad de La República, Montevideo, Uruguay; ^8^ Ministerio de Ganadería, Agricultura y Pesca, Montevideo, Uruguay; ^9^ Departamento de Bioquímica y Genómica Microbianas, Instituto de Investigaciones Biológicas Clemente Estable, Montevideo, Uruguay; ^10^ Museo y Jardín Botánico “Prof. Atilio Lombardo”, Intendencia de Montevideo, Montevideo, Uruguay; ^11^ Centro Universitario Regional Este, Universidad de La República, Rocha, Uruguay; ^12^ Uruguayan Barcode of Life initiative, Montevideo, Uruguay

**Keywords:** Bold System, taxonomy, uruguayan flora, molecular markers, *rbcL*, *trnH-psbA*, *trnL (UAA)*, *ITS2*

## 1 Introduction

The loss of biodiversity has accelerated during the Anthropocene era, matching rates seen in previous mass extinctions (Rockström et al., 2009), resulting in unprecedented environmental changes (IPBES, 2021). In this scenario, it is crucial to make progress in effectively describing, documenting, and classifying living species. Acknowledging this urgency, the Convention on Biological Diversity (CBD) has highlighted the essential role of taxonomy in achieving these goals (CBD, 2021). However, human resources with expertise in taxonomy are increasingly scarce and often face intricate challenges like identifying cryptic species and larval stages (Thomsen and Willerslew, 2015). These challenges have sparked a need for alternative approaches in biodiversity description and monitoring. In response, Barcodes of Life (BoL) have emerged to identify organisms through genomic approaches utilizing specific DNA regions and standardized protocols (Hebert et al., 2003). The potential of this strategy has been demonstrated on a global scale as a powerful tool, with widespread applications including natural resource conservation, safeguarding endangered species, agricultural pest management, disease vector identification, water quality surveillance, verification of health and food products, forensic entomology, as well as medicinal plant identification, among others (Consortium for the Barcode of Life, 2007).

DNA techniques require the meticulous selection of suitable barcoding regions, involving the identification of standardized loci that can be systematically and reliably sequenced across extensive and diverse taxa. This enables the generation of easily comparable data that facilitates species distinction (Hollingsworth et al., 2011). In animals, *cytochrome oxidase I* (*COI*) is the standard DNA barcode, showing notable success in precisely identifying species (Hebert et al., 2003). However, identifying an equivalent DNA barcode for plants remains challenging due to the absence of a single, appropriate locus for species delimitation (Hebert et al., 2016). Plant DNA barcoding has been controversial due to the presence of hybridization, introgression processes, and polyploidy (Hebert et al., 2016). The slow evolutionary rate in plant mitochondrial genomes rules out *COI* as a universal plant barcode (Hollingsworth et al., 2011). Instead, several chloroplast and nuclear molecular markers like *rbcL*, *matK*, *trnH-psbA*, *trnL (UAA)*, and *ITS* have been explored as viables alternatives (Taberlet et al., 2007; Hollingsworth et al., 2011; Marcial-Quino et al., 2015). Given the inadequate species discrimination in certain plant groups with a single molecular marker, a combination of markers has been proposed (Hollingsworth et al., 2011).

Another central aspect for taxa recognition using molecular markers is the need for a comprehensive reference database of barcode DNA sequences encompassing a wide range of species (Hollingsworth et al., 2011; Tnah et al., 2019). The quantity of sequences accessible for a specific locus impacts its discriminatory power (Hollingsworth et al., 2011). Establishing a robust public sequence repository is essential for assessing multiple potential loci for barcoding and their combination (Hollingsworth et al., 2011; Kolter and Gemeinholzer, 2021).

Based on these principles, the Consortium for the Barcode of Life (CBoL; www.barcodeoflife.org) was established and is collaboratively building the Barcode of Life Data Systems (BOLD, Ratnasingham and Hebert, 2007; www.boldsystems.org). BOLD is a global open access repository of DNA barcodes, but unlike other public genomic repositories such as GenBank (NCBI), EMBL, and DDBJ, it is specifically tailored for biodiversity and species identification through DNA barcoding (Borisenko et al., 2009), linking morphological and distributional data with DNA sequences. It also supports broader taxonomic initiatives such as the Global Taxonomic Initiative (GTI) for the Convention on Biological Diversity (CBD) and the Global Biodiversity Information Facility (GBIF) (Costa and Antunes, 2012).

Among the vast array of over 375,000 documented vascular plant species worldwide (Hong et al., 2022), only 115,768 are registered within BOLD’s database with a total of 380,813 public records (BOLD, 22/04/2024). This underscores the pressing necessity for substantial contributions from diverse researchers, institutions, and national networks to enhance the comprehensiveness of this database.

In recent years, Uruguay has experienced a decline in biodiversity, a global phenomenon impacting ecosystems worldwide. The dramatic shifts in land use since the 1990s, particularly marked by an increase in the area of tree plantations (the highest in South America) and agriculture (specifically soybean and rice), underscore the urgent need for effective advances in documenting and categorizing the country’s biodiversity (Paruelo et al., 2006; Baeza and Paruelo, 2020). Currently, vascular plants in Uruguay remains largely unexplored in terms of barcode sequence availability. The BOLD platform holds 1,131 public records for Uruguayan organisms, representing 323 species, with only 24 vascular plant species documented (searched on 22/3/2024), which accounts for less than 1% of native species reported for the country (Bonifacino et al., 2019). As a way of filling this void of DNA barcode sequences among the Uruguayan biodiversity, a major initiative is underway to establish a DNA barcode national network. During October 2018 the “Barcode of Life Training Course: example from native flora” was held, under the call for the program “Filling the capacity gap for the application of DNA technologies in taxonomy -GTI trainings/workshops driven by the trained trainers.” This project had two main objectives: i) to train undergraduate and postgraduate students, as well as technicians from public and private institutions; to conduct workshops with vascular plant specialists to list key species of the Uruguayan flora; and ii) to coordinate with key decision makers regarding national biodiversity and scientific collections, with the intention of establishing and maintaining a national DNA barcode network in the near future.

Our objective was to produce a first batch of DNA barcode sequences for the vascular plants of Uruguay, focusing on species not previously documented in BOLD that hold cultural significance in Uruguay due to their agricultural, medicinal and horticultural uses, employing four commonly used molecular markers for plants (*rbcL*, *trnH-psbA*, *trnL (UAA)*, and *ITS2*).

## 2 Materials and methods

### 2.1 Species selection

In order to select the plant species to be analyzed, two meetings were organized with experts in grassland ecosystems, livestock production in natural grasslands, and native forests, as well as invited taxonomists. The aim of these meetings was to compile an initial list of key species representing Uruguayan plant biodiversity, based on their productive value, symbolic importance or conservation status.

### 2.2 Sample collection and storage

A total of 51 samples, representing 50 vascular plant species were collected in different regions of Uruguay: grasslands of Rivera and Río Negro departments; the greenhouse of the Instituto Nacional de Semillas (INASE) in Canelones department; the gardens of the Museo Jardín Botánico Profesor Atilio Lombardo (MJBAL-IMM), the Facultad de Agronomía of the Universidad de la República (FAgro-Udelar), and the Instituto de Investigaciones Biológicas Clemente Estable (IIBCE-MEC) in Montevideo department (Table 1).

**TABLE 1:** Specimens reported in this study. For each sample, the following information is listed: process identification in BOLD, species name and taxonomy, institution where the voucher is deposited (Universidad de la República, Facultad de Agronomía, Bernardo Rosengurtt Herbarium = MVFA, Museo y Jardin Botánico Prof. Atilio Lombardo = MVJB, Instituto de Investigaciónes Biológicas Clemente Estable = IIBCE), whether the species has been previously documented in BOLD, if the documented specimens in BOLD for the species were collected in Uruguay, and whether sequencing was successful for each of the four markers used in this study. In the cases where the origin of the samples in BOLD is missing a question mark is added.

							BOLD		Molecular markers			
Process ID	Species	Phylum	Class	Order	Family	Institution Storing	Species documented	species with specimens collected in Uruguay	*trn*H-*psb*A	*trn*L	*rbc*L	ITS2
MNATU001-18	*Nassella neesiana* (Trin. and Rupr.) Barkworth	Magnoliophyta	Liliopsida	Poales	Poaceae	MVFA	yes	yes	yes	yes	yes	no
MNATU002-18	*Psidium cattleyanum* Sabine	Magnoliophyta	Magnoliopsida	Myrtales	Myrtaceae	MVJB	yes	no	no	yes	no	yes
MNATU003-18	*Parapiptadenia rigida* (Benth.) Brenan	Magnoliophyta	Magnoliopsida	Fabales	Fabaceae	MVFA	yes	no	yes	no	yes	no
MNATU004-18	*Lantana camara L.*	Magnoliophyta	Magnoliopsida	Lamiales	Verbenaceae	MVFA	yes	no	yes	yes	yes	yes
MNATU005-18	*Citharexylum montevidense* Moldenke	Magnoliophyta	Magnoliopsida	Lamiales	Verbenaceae	MVFA	yes	no	yes	yes	yes	yes
MNATU007-18	*Allophyllus edulis* (A.St.-Hil., A.Juss. and Cambess.) Hieron. ex Niederl.	Magnoliophyta	Magnoliopsida	Sapindales	Sapindaceae	MVFA	yes	no	yes	yes	yes	yes
MNATU008-18	*Acca sellowiana* (O.Berg)Burret	Magnoliophyta	Magnoliopsida	Myrtales	Myrtaceae	MVJB	yes	no	yes	yes	yes	yes
MNATU009-18	*Ficus luschnathiana* Miq.	Magnoliophyta	Magnoliopsida	Rosales	Moraceae	MVJB	yes	no	yes	yes	yes	yes
MNATU010-18	*Azara uruguayensis* (Speg.) Sleumer	Magnoliophyta	Magnoliopsida	Malpighiales	Salicaceae	MVJB	no	no	yes	yes	yes	yes
MNATU011-18	*Myrcianthes pungens* (O.Berg) D.Legrand	Magnoliophyta	Magnoliopsida	Myrtales	Myrtaceae	MVJB	yes	no	yes	yes	yes	yes
MNATU012-18	*Eugenia uruguayensis* Cambess.	Magnoliophyta	Magnoliopsida	Myrtales	Myrtaceae	MVJB	yes	no	yes	yes	yes	yes
MNATU014-18	*Acanthosyris spinescens* Griseb.	Magnoliophyta	Magnoliopsida	Santalales	Santalaceae	MVJB	yes	no	yes	yes	yes	yes
MNATU015-18	*Smilax campestris* Griseb.	Magnoliophyta	Liliopsida	Liliales	Smilacaceae	MVJB	no	no	no	yes	yes	no
MNATU016-18	*Vitex megapotamica* (Spreng.) Moldenke	Magnoliophyta	Magnoliopsida	Lamiales	Lamiaceae	MVJB	yes	no	yes	yes	yes	yes
MNATU017-18	*Myrsine laetevirens* (Mez) Arechav.	Magnoliophyta	Magnoliopsida	Ericales	Primulaceae	MVJB	no	no	yes	yes	yes	yes
MNATU018-18	*Berberis laurina* Thunb.	Magnoliophyta	Magnoliopsida	Ranunculales	Berberidaceae	MVJB	no	no	yes	yes	yes	yes
MNATU020-18	*Vachellia caven (Molina)* Seigler and Ebinger	Magnoliophyta	Magnoliopsida	Fabales	Fabaceae	MVJB	yes	no	yes	yes	yes	no
MNATU022-18	*Labatia salicifolia* (Spreng.) Mart.	Magnoliophyta	Magnoliopsida	Ericales	Sapotaceae	IIBCE	no	no	no	yes	yes	yes
MNATU023-18	*Mnesithea selloana* (Hack.) de Koning and Sosef	Magnoliophyta	Liliopsida	Poales	Poaceae	MVFA	no	no	yes	yes	yes	no
MNATU024-18	*Paspalum plicatulum* Michx.	Magnoliophyta	Liliopsida	Poales	Poaceae	MVFA	no	no	yes	yes	yes	yes
MNATU025-18	*Panicum hians* Elliott	Magnoliophyta	Liliopsida	Poales	Poaceae	MVFA	no	no	yes	yes	yes	yes
MNATU026-18	*Tridens brasiliensis* (Steud.) Nees ex Parodi	Magnoliophyta	Liliopsida	Poales	Poaceae	MVFA	no	no	yes	yes	no	yes
MNATU027-18	*Schizachyrium microstachyum (Ham.) Roseng., B.R.Arrill. and Izag.*	Magnoliophyta	Liliopsida	Poales	Poaceae	MVFA	no	no	yes	yes	yes	no
MNATU028-18	*Mimosa ramulosa* Benth.	Magnoliophyta	Magnoliopsida	Fabales	Fabaceae	MVFA	no	no	yes	yes	yes	no
MNATU029-18	*Mimosa magentea* Izag. and Beyhaut	Magnoliophyta	Magnoliopsida	Fabales	Fabaceae	MVFA	no	no	yes	yes	yes	no
MNATU030-18	*Mimosa schleidenii Herter*	Magnoliophyta	Magnoliopsida	Fabales	Fabaceae	MVFA	no	no	no	yes	yes	no
MNATU031-18	*Lithraea brasiliensis* Marchand	Magnoliophyta	Magnoliopsida	Sapindales	Anacardiaceae	MVJB	no	no	yes	yes	yes	yes
MNATU032-18	*Myrrhinium octandrum* (Benth) Mattos	Magnoliophyta	Magnoliopsida	Myrtales	Myrtaceae	MVJB	yes	no	yes	yes	yes	yes
MNATU033-18	*Adiantum thalictroides* Schltdl.	Pteridophyta	Polypodiopsida	Polypodiales	Pteridaceae	MVJB	no	no	yes	no	no	no
MNATU034-18	*Axonopus argentinus* Parodi	Magnoliophyta	Liliopsida	Poales	Poaceae	MVJB	yes	no	yes	yes	yes	yes
MNATU035-18	*Schinus lentiscifolia* Marchand	Magnoliophyta	Magnoliopsida	Sapindales	Anacardiaceae	MVJB	no	no	yes	no	yes	yes
MNATU036-18	*Myrsine parvifolia* A.DC.	Magnoliophyta	Magnoliopsida	Ericales	Primulaceae	MVJB	no	no	yes	yes	yes	yes
MNATU038-18	*Duranta repens L.*	Magnoliophyta	Magnoliopsida	Lamiales	Verbenaceae	MVJB	yes	no	no	yes	yes	yes
MNATU039-18	*Jodina rhombifolia (Hook. and Arn.) Hook. and Arn. ex Reissek*	Magnoliophyta	Liliopsida	Liliales	Smilacaceae	MVJB	no	no	no	yes	yes	yes
MNATU040-18	*Enterolobium contortisiliquum* (Vell.) Morong.	Magnoliophyta	Magnoliopsida	Fabales	Fabaceae	MVJB	yes	?	yes	yes	yes	no
MNATU043-18	*Parapiptadenia rigida* (Benth.) Brenan	Magnoliophyta	Magnoliopsida	Fabales	Fabaceae	MVJB	yes	?	yes	yes	no	no
MNATU044-18	*Pyracantha coccinea* M.Roem.	Magnoliophyta	Magnoliopsida	Rosales	Rosaceae	MVJB	yes	no	no	yes	yes	yes
MNATU045-18	*Phoenix canariensis* H.Wildpret	Magnoliophyta	Liliopsida	Arecales	Arecaceae	MVJB	yes	no	no	yes	yes	no
PASUY001-18	*Nassella filiculmis* (Delile) Barkworth	Magnoliophyta	Liliopsida	Poales	Poaceae	MVFA	yes	no	yes	yes	yes	yes
PASUY002-18	*Sorghastrum pellitum* Parodi	Magnoliophyta	Liliopsida	Poales	Poaceae	MVFA	no	no	yes	yes	yes	yes
PASUY003-18	*Jarava filifolia* (Nees) Ciald	Magnoliophyta	Liliopsida	Poales	Poaceae	MVFA	no	no	yes	yes	yes	yes
PASUY004-18	*Chascolytrum subaristatum* (Lam.) Desv.	Magnoliophyta	Liliopsida	Poales	Poaceae	MVFA	no	no	yes	yes	yes	yes
PASUY005-18	*Bothriochloa laguroides* (DC.) Herter	Magnoliophyta	Liliopsida	Poales	Poaceae	MVFA	yes	no	yes	yes	yes	yes
PASUY006-18	*Eragrostis retinens* Hack. and Arechav.	Magnoliophyta	Liliopsida	Poales	Poaceae	MVFA	no	no	no	yes	yes	yes
PASUY007-18	*Paspalum urvillei* Steud.	Magnoliophyta	Liliopsida	Poales	Poaceae	MVFA	yes	no	yes	yes	yes	yes
PASUY008-18	*Chloris canterae* Arechav.	Magnoliophyta	Liliopsida	Poales	Poaceae	MVFA	yes	yes	yes	yes	yes	yes
PASUY009-18	*Panicum bergii* Arechav.	Magnoliophyta	Liliopsida	Poales	Poaceae	MVFA	yes	no	yes	yes	yes	yes
PASUY010-18	*Sporobolus indicus* (L.)R.Br.	Magnoliophyta	Liliopsida	Poales	Poaceae	MVFA	yes	no	no	yes	no	no
PASUY011-18	*Paspalum notatum* Flüggé	Magnoliophyta	Liliopsida	Poales	Poaceae	MVFA	yes	no	yes	yes	yes	yes
PASUY012-18	*Axonopus affinis* Chase	Magnoliophyta	Liliopsida	Poales	Poaceae	MVFA	no	no	yes	yes	yes	no
PASUY013-19	*Myrcianthes cisplatensis* (Cambess.) O.Berg.	Magnoliophyta	Magnoliopsida	Myrtales	Myrtaceae	MVJB	yes	yes	yes	yes	yes	yes

Vascular plants specimens sampled belonged to 13 orders and 16 families. The three orders best represented in the sample were Poales (37.25%), Fabales (13.73%) and Myrtales (11.76%). This includes 23 species not previously documented in BOLD and 23 species documented in BOLD, but without specimens collected in Uruguay (Table 1).

**FIGURE 1:**
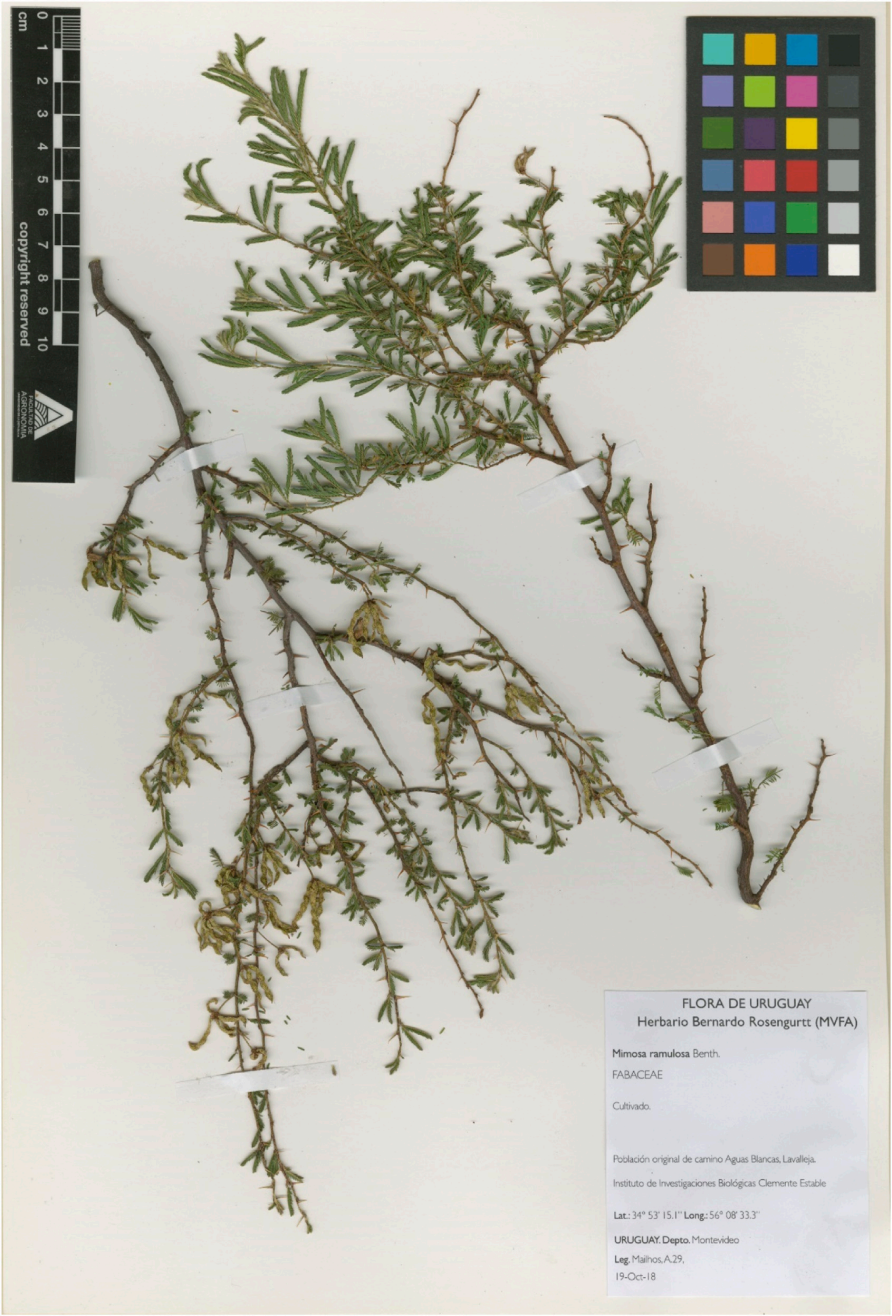
Herbarium voucher. *A Mimosa ramulosa* Benth. specimen, submitted to BOLD with the Process ID MNATU028-18 and deposited in MVFA herbarium.

Standardized procedures were followed to obtain herbarium samples (Bridson and Forman, 1992; Seshagirirao et al., 2016) and leaf or stem tissue for DNA extraction (Wilkie et al., 2013). A high-quality image of the vouchers was obtained using an EPSON Expression 10000XL (Figure 1). Tissue samples for DNA isolation were stored in silica gel until dry. Specimens were deposited in MVJB, MVFA and IIBCE-MEC herbaria (Table 1). Herbarium acronyms according to Thiers (updated continuously).

### 2.3 Laboratory procedures

DNA extractions were conducted from dried unexpanded young leaves, following Fazekas et al. (2012) protocol. FastPrep®- 24 (MP Biomedicals) and lysing matrix beads were used for sample lysis. Afterwards, four DNA barcode fragments were amplified, three from chloroplast: *rbcL*, *trnH-psbA* and *trnL(UUA)* (Fay et al., 1997; Taberlet et al., 2007; Guo et al., 2011) and one nuclear (*ITS2*) (Chen et al., 2010) (Supplementary Material). Each PCR reaction contained 1X Buffer (Invitrogen), 1.5 mM MgCl (Invitrogen), 0.04 U Taq polymerase (Invitrogen), 0.05 mM each dNTP, 0.2 mg/mL BSA and 0.5 µM forward and reverse primers in a final volume of 20 µL. The thermal profile consisted of an initial denaturalization step of 94°C for 3 min, followed by 35 cycles of 94°C for 30 s, annealing for 30 s, (annealing temperatures for each genetic marker are provided in the Supplementary Material), and 72°C for 1 min, a final extension step of 72°C for 15 min were inserted. PCR amplifications were conducted independently for each molecular marker and negative controls were included on each PCR and extraction reactions. Moreover, PCRs and DNA extractions were carried out in different rooms to avoid contaminations.

Amplification success was confirmed through 1% agarose electrophoresis using GoodView^TM^ nucleic acid stain (SBS Genetech Co., Ltd, Beijing). PCR products were purified using an enzymatic method with 0.8 U/μL of Exonuclease I and 0.2 U/μL of Thermosensitive Alkaline Phosphatase (FastAP) (ThermoFisher Scientific^TM^). The conditions for purifications were 37°C for 1 h and 30 min followed by 5 min at 75°C. The amount of purified PCR products was measured by NanodropTMND-1000 UV-vis Spectrophotometer (Nano-Drop Techonologies, Inc., Wilmington, DE, United States). Forward and reverse sequencing was conducted on ABI3500 (Applied Biosystems) in Institute Pasteur de Montevideo and Applied Biosystems 3730XL in Macrogen Inc.

### 2.4 Data treatment

Every DNA barcode sequence was analyzed by eye and alignment was built by MUSCLE using Mega5 (Tamura et al., 2011). Sequences obtained for each locus and specimen were compared with public sequences through BLAST against GenBank database (Altschul et al., 1990) to confirm sequence identity. The data generated for each sample was submitted to Barcode of Life Data System (BOLD, http://www.boldsystems.org).

## 3 Conclusion

The sample collection and laboratory procedures employed in this study proved to be accurate for the plant DNA barcoding protocol, resulting in 171 sequences from four loci for 50 vascular plant species. The selection of appropriate markers for taxa identification involves evaluating primer universality, sequence quality, and discriminatory power (Hollingsworth et al., 2011). Our study assessed the first two criteria, indicating that, in agreement with previous findings for land plants (Hollingsworth et al., 2011), *trnL (UAA)* and *rbcL* are the most suitable loci for the plant groups examined (Table 1). Plastid loci have been widely used in phylogenetic analyses, presenting traits similar to those seen in the mitochondrial genome of animals (Kress et al., 2005; Chase et al., 2007). Notably, the *ITS2* amplification rates observed in our samples were comparatively low, diverging from previous studies (Chase et al., 2007; Chen et al., 2010). Finally, setting a comprehensive public database of sequences from several candidate loci for a wide variety of species is crucial not only for the evaluation of barcode candidates and their combinations (Hollingsworth et al., 2011), but also for an accurate species differentiation (Hollingsworth et al., 2011; Tnah et al., 2019). Our contribution aims to lay the groundwork for a robust database to further research in biodiversity and facilitate taxonomic identification.

This work marks the first step of the Uruguayan Barcode of Life Initiative (BoL-UY), providing essential insights into vascular plants with data from three chloroplast loci and one nuclear locus. Embracing the DNA barcoding protocol, voucher images of specimens remain accessible and linked to sequences for each locus in BOLD. The data generated holds promise for future research venues aimed at identifying the optimal loci and their most effective combinations for vascular plants DNA barcoding.

## Data Availability

The data presented in the study are deposited in the Barcode of Life Data System (www.boldsystems.org), accession number are listed in Table 1 as ProcessID.
