# Supplementary material for: Exploring biodiversity of Uruguayan vascular plants through DNA barcoding
Source: Front Genet. 2024 Aug 21;15:1435592. doi: 10.3389/fgene.2024.1435592 (PMC11463761; doi:10.3389/fgene.2024.1435592)
Supplement: Supplementary file 1 [file Table1.DOCX]

**Supplementary Table 1**: Primers used for vascular plants barcode. For each barcode fragment the following information is listed: primers sequences, annealing temperature (Ta), amplified fragment length in base pair (bp), number of sequences obtained (N) and the bibliographic references. The primers with an asterisk (*) amplifies a nuclear fragment.

| Primer | Primer Sequence (5´- 3´) | Ta (°C) | Fragment length (bp) | N | Reference |
| --- | --- | --- | --- | --- | --- |
| rbcL 1 Forward | ATGTCACCACAAACAGAAAC | 50 | 600 | 48 | Fay et al. 1997 |
| rbcL 724 Reverse | TCGCATGTACCTGCAGTAGC |  |  |  |  |
| trnH 05 Reverse | GTTATGCATGAACGTAATGCTC | 55 | 500 | 52 | Guo et al. 2011 |
| psbA 3 Forward | CGCGCATGGTGGATTCACAATCC |  |  |  |  |
| trnLc Forward | CGAAATCGGTAGACGCTACG | 50 | 500 | 50 | Taberlet et al. 2007 |
| trnLd Reverse | GGGGATAGAGGGACTTGAAC |  |  |  |  |
| ITS2_S2F Forward* | ATGCGATACTTGGTGTGAAT | 56 | 500 | 43 | Chen et al. 2010 |
| ITS2_S3R Reverse* | GACGCTTCTCCAGACTACAAT |  |  |  |  |
